# Duloxetine for the long-term treatment of Major Depressive Disorder in patients aged 65 and older: an open-label study

**DOI:** 10.1186/1471-2318-4-11

**Published:** 2004-12-07

**Authors:** Madelaine M Wohlreich, Craig H Mallinckrodt, John G Watkin, Donald P Hay

**Affiliations:** 1 Lilly Research Laboratories, Eli Lilly and Company, Indianapolis, IN 46285, USA

## Abstract

**Background:**

Late-life depression is a common, chronic and recurring disorder for which guidelines recommend long-term therapy. The safety and efficacy of duloxetine for the treatment of major depressive disorder (MDD) were evaluated using data from elderly patients (age ≥ 65 years; n = 101) who participated in a large, multinational, open-label study.

**Methods:**

Patients meeting DSM-IV criteria for MDD received duloxetine 80 mg/d (40 mg twice daily (BID)) to 120 mg/d (60 mg BID) for up to 52 weeks. Efficacy measures included the Clinical Global Impression of Severity (CGI-S) scale, the 17-item Hamilton Rating Scale for Depression (HAMD_17_), the Beck Depression Inventory-II (BDI-II), the Patient Global Impression of Improvement (PGI-I) scale, and the Sheehan Disability Scale (SDS). Safety and tolerability were evaluated using discontinuation rates, spontaneously reported adverse events, and changes in vital signs, ECG, and laboratory analytes.

**Results:**

Mean changes in HAMD_17 _total score at Weeks 6, 28, and 52 were -13.0, -17.4 and -17.5 (all p-values <.001). Significant improvement (p < .001) in both clinician- (CGI-S) and patient-rated (PGI-I) measures of improvement were observed at Week 1 and sustained throughout the study. Observed case response rates at Weeks 6, 28, and 52 were 62.9%, 84.9%, and 89.4%, respectively, while the corresponding rates of remission were 41.4%, 69.8%, and 72.3%. Adverse events led to discontinuation in 27 (26.7%) patients. Treatment-emergent adverse events reported by >10% of patients included dizziness, nausea, constipation, somnolence, insomnia, dry mouth, and diarrhea. Most events occurred early in the study. Mean changes at endpoint in blood pressure and body weight were less than 2.0 mm Hg, and -0.1 kg, respectively.

**Conclusions:**

In this open-label study, duloxetine was effective, safe, and well tolerated in the long-term treatment of MDD in patients aged 65 and older.

## Background

Late-life depression is a common and disabling condition which represents a substantial public health concern [1]. The prevalence of major depressive disorder (MDD) in the community-dwelling elderly population is estimated at 1–3%, with depressive symptoms being present in approximately 15% [2]. The rate of occurrence of MDD is even higher among institutionalized older patients. In long-term care patients the incidence has been estimated to be 12% to 25%, with subsyndromal depressive symptoms present in an additional 18% to 30% [3].

Despite advances in available antidepressant treatments, limitations still exist in both efficacy and safety. Tricyclic antidepressants (TCAs) generally provide robust efficacy, but a number of side effects associated with this class of medications are of particular concern in older patients (e.g. anticholinergic adverse events, orthostatic hypotension, and sedation). Selective serotonin reuptake inhibitors (SSRIs) have provided an improved tolerability profile compared to the TCAs through lower rates of adverse events, and substantially lower toxicity in overdose [4]. Furthermore, SSRIs do not appear to exhibit age-related increases in occurrence of adverse events [5]. However, these newer selective antidepressants appear, in general, to achieve equivalent or lower remission rates compared with the older tricyclics [6].

Duloxetine is a potent dual reuptake inhibitor of serotonin (5-HT) and norepinephrine (NE) [7]. The efficacy of duloxetine in the acute treatment of MDD has been established in randomized, double-blind, placebo-controlled studies in patients aged 18 and older [8-11]. A subsequent post-hoc analysis of efficacy data from these studies, focusing upon those patients aged 55 and older receiving once-daily duloxetine (60 mg), supported the findings in the general patient population [12].

The safety and tolerability of duloxetine have also been demonstrated under double-blind conditions. In placebo-controlled trials of duloxetine in patients aged 18 and older (doses from 40 – 120 mg/d) the most frequently reported adverse events were nausea, headache, dry mouth, fatigue, insomnia, and dizziness, while the overall safety profile of duloxetine was comparable to that of available SSRI medications [11]. A comparable safety and tolerability profile was observed following a post-hoc analysis of data from those patients aged 55 and older, including a low incidence of cardiovascular adverse events and minimal effects upon blood pressure and heart rate [12].

However, these acute placebo-controlled trials of duloxetine were of 9 weeks duration or less. An NIH consensus panel has recommended that geriatric patients be given continuing antidepressant treatment for at least 6 months for a first episode and for at least 1 year for recurrent episodes [13], while some investigators suggest that maintenance treatment in the elderly be extended to 2 years [14]. In order to evaluate the long-term tolerability, safety, and efficacy of duloxetine, a one-year open-label trial in depressed patients was undertaken. This report examines the subset of patients aged 65 and older who participated in the study. While patients in this study received doses of 80 mg/d or 120 mg/d, it should be noted that the approved dose range for duloxetine for the treatment of MDD is 40–60 mg/d.

## Methods

### Study design

This was a 52-week, open-label, single-arm study of outpatients (aged ≥ 18 years) meeting *Diagnostic and Statistical Manual of Mental Disorders, 4th Edition *(DSM-IV) [15] criteria for MDD. The study included a total of 1279 patients at 52 investigative sites in Argentina, Brazil, Canada, Columbia, Mexico, the United States, and Venezuela. The primary objective of the study was to evaluate the safety of duloxetine (80 or 120 mg/d given as two equal doses per day, i.e. 40 to 60 mg BID) for up to 52 weeks. During the first week of therapy, all patients received duloxetine 40 mg BID. Patients unable to tolerate 40 mg BID could have their dose decreased to 20 mg BID, but were required to increase the dose to 40 mg BID at Week 2. Patients unable to tolerate 40 mg BID were discontinued from the study. During the remainder of the study, the patient's dose could be adjusted up to 60 mg BID or down to 40 mg BID, based upon the physician's clinical evaluation of tolerability and efficacy.

This report focuses upon data taken from the subset of patients aged 65 years and older (n = 101) within the larger study described above.

### Patients

The study protocol was approved by the ethics committee at each site in accordance with the principles of the Declaration of Helsinki. All patients provided written informed consent prior to the administration of any study procedures or study drug. All patients were required to have a Clinical Global Impression of Severity (CGI-S) score ≥ 3 at the screening and baseline study visits.

Patients were excluded for the following reasons: a previous or current diagnosis of schizophrenia, schizophreniform disorder, schizoaffective disorder, or bipolar disorder; presence of an Axis II disorder that would interfere with protocol compliance; serious medical illness; taking benzodiazepines on a daily basis for ≥ 2 weeks prior to enrollment; a history of substance dependence within the last year; or a positive urine drug screen. Subjects judged to be at risk for suicide were also excluded.

### Concomitant medications

Patients were not permitted to receive other antidepressant, antimanic, or antipsychotic agents during the study. Episodic use (≤ 3 consecutive days, and no more than 100 total days) of benzodiazepines was permitted. The use of benadryl, chloral hydrate, cough and cold medications, and narcotics, was allowed on an episodic basis only. Subjects were permitted to take antihypertensives, antiarrhythmics, antibiotics, and multivitamins among other medications while in the study.

### Efficacy measures

Efficacy was assessed using the CGI-S scale [16] (*a priori *specified as the primary outcome), the HAMD_17 _total score [17], HAMD_17 _subscales (core – Items 1, 2, 3, 7, and 8; Maier – Items 1, 2, 7, 8, 9, and 10; anxiety/somatization – Items 10, 11, 12, 13, 15, and 17; retardation – Items 1, 7, 8, and 14; sleep – Items 4, 5, and 6), the Beck Depression Inventory-II (BDI-II) [18], and the Patient Global Impression of Improvement (PGI-I) scale [16]. Patient-rated quality of life was evaluated using the Sheehan Disability Scale (SDS) [19], which is a composite of 3 self-rated 10-point Likert response subscales (0 = no disability, 1–3 = mild, 4–6 = moderate, 7–9 = marked, 10 = extreme) to assess work, family, and social functioning during the past month. All outcomes were assessed at Weeks 6, 28, and 52, or upon early discontinuation, except for PGI-I and CGI-S scales which were collected at all visits. Patients were defined as *responders *if they had a decrease from baseline of at least 50% in HAMD_17 _total score. Patients were defined as *remitters *if they had a HAMD_17 _total score ≤ 7.

### Safety measures

Safety measures included spontaneously reported adverse events, serious adverse events (events that led to outcome of death, inpatient hospitalization, cancer, severe or permanent disability, congenital abnormality, or life-threatening condition), vital signs, electrocardiograms (ECGs), and laboratory analyses. Adverse events and vital signs were collected at each visit.

Lilly reference ranges were used to define limits for abnormal laboratory values [20], and potentially clinically significant (PCS) changes in selected laboratory analytes [21]. PCS changes in blood pressure were defined as follows:

(i) Low supine (or standing) systolic BP: ≤ 90 mm Hg **and **a decrease from baseline of ≥ 20 mm Hg;

(ii) High supine (or standing) systolic BP: ≥ 180 mm Hg **and **an increase from baseline of ≥ 20 mm Hg

(iii) Low supine (or standing) diastolic BP: ≤ 50 mm Hg **and **a decrease from baseline of ≥ 15 mm Hg;

(iv) High supine (or standing) diastolic BP: ≥ 105 mm Hg **and **an increase from baseline of ≥ 15 mm Hg.

Patients were considered hypertensive at baseline if they had a historical diagnosis, secondary condition, or adverse event at the baseline visit consistent with a clinical diagnosis of hypertension or high blood pressure.

ECGs were collected at baseline and Weeks 4, 28, 52 or at early discontinuation. Patients at 2 sites in Mexico and 1 site in Columbia also had ECGs over-read by a cardiologist at a central location. For these ECGs, QT intervals were corrected (QTc) using Fridericia's correction (QTcF). All other patients had ECGs read by the site for classification as either normal or abnormal. Limits for PCS QTc values were an increase in QTcF of ≥ 30 msec and any postbaseline value ≥ 450 msec for males or ≥ 470 msec for females [22].

### Statistical analyses

Mean changes from baseline to last observation in laboratory analytes, vital signs, and ECG intervals were assessed using ANOVA with models that included investigator. Longitudinal mean changes and categorical changes (temporal patterns) were assessed via a likelihood-based repeated measures approach. Models for mean changes included investigator, visit, baseline value, and baseline-by-visit interaction.

Mean change in CGI-S score was compared between younger (age <65) and elderly (age ≥ 65) patients using the repeated measures analysis as previously described, with age group and age group-by-visit interaction added to the model. Differences between young and elderly patients in rates of treatment emergent adverse events were assessed using Fisher's exact test.

## Results

### Patient disposition

This report was based on data from 101 patients aged 65 and older. The oldest patient was 87 years of age, while the median age was 70. Patient characteristics at baseline are summarized in Table 1.

**Table 1:** Summary of patient demographics and psychiatric history^a^

	**Duloxetine, 80–120 mg/d^† ^(n = 101)**
**Gender**, n (%)	
Female	72 (71.3)
Male	29 (28.7)
**Age**, y	71.9 (5.4)
**Age range**, y	65 – 87
**Weight**, kg	66.5 (14.5)
**Ethnicity**, n (%)	
Caucasian	43 (42.6)
Hispanic	55 (54.5)
Other	3 (3.0)
**Age at onset**, y	63.5 (13.3)
**Current duration**, wks	86.0 (161.0)
**Number of previous episodes**	1.1 (2.1)
**Duration of last episode**, wks	57.6 (110.2)

a. Listed as mean (SD) unless otherwise stated.

† Administered as 40 mg BID or 60 mg BID

### Efficacy

Mean changes from baseline for all efficacy outcomes were highly significant (p < .001, t-test for mean change) at all assessment times (Table 2). In the case of CGI-S and PGI-I scales, significant improvements were observed at Week 1 and at all subsequent visits (p < .001, t-test for mean). Observed case response rates at Weeks 6, 28, and 52 were 62.9% (44/70), 84.9% (45/53), and 89.4% (42/47), respectively, while the corresponding rates of remission were 41.4% (29/70), 69.8% (37/53), and 72.3% (34/47), respectively.

**Table 2:** Efficacy outcome measures

**Outcome measure**	**Mean baseline score**	**Mean change (SE)**		
		
		**Week 6**	**Week 28**	**Week 52**
**CGI-Severity**	4.51	-2.08 (0.11)**	-2.93 (0.12)**	-3.15 (0.12)**
**PGI-Improvement**	N/A	2.33 (0.14)**	1.83 (0.16)**	1.84 (0.16)**
**HAMD_17 _Total Score**	21.8	-13.0 (0.7)**	-17.4 (0.8)**	-17.5 (0.8)**
Anxiety subscale	6.70	-3.46 (0.30)**	-4.89 (0.33)**	-4.90 (0.34)**
Core subscale	8.83	-5.65 (0.33)**	-7.50 (0.36)**	-7.61 (0.38)**
Maier subscale	10.7	-6.64 (0.38)**	-8.90 (0.42)**	-9.06 (0.44)**
Retardation subscale	7.84	-4.49 (0.27)**	-6.58 (0.30)**	-6.49 (0.31)**
Sleep subscale	3.68	-2.34 (0.21)**	-2.84 (0.23)**	-2.83 (0.24)**
HAMD_17 _Item 1	2.64	-1.73 (0.11)**	-2.30 (0.13)**	-2.30 (0.13)**
HAMD_17 _Item 3	0.74	-0.58 (0.06)**	-0.59 (0.06)**	-0.61 (0.06)**
**BDI-II Total Score**	29.5	-15.8 (1.0)**	-22.3 (1.1)**	-22.0 (1.1)**
**Sheehan Disability Scale**				
Work item	6.91	-3.01 (0.32)**	-4.60 (0.37)**	-4.27 (0.39)**
Family item	6.82	-3.63 (0.32)**	-4.88 (0.35)**	-4.95 (0.37)**
Social item	7.27	-3.45 (0.34)**	-4.57 (0.38)**	-4.85 (0.40)**

** p < .001 from t-test for mean change

CGI-Severity = Clinical Global Impression of Severity; PGI-Improvement = Patient Global Impression of Improvement; HAMD_17 _= 17-item Hamilton Rating Scale for Depression; BDI-II = Beck Depression Inventory-II

**Figure 1:**
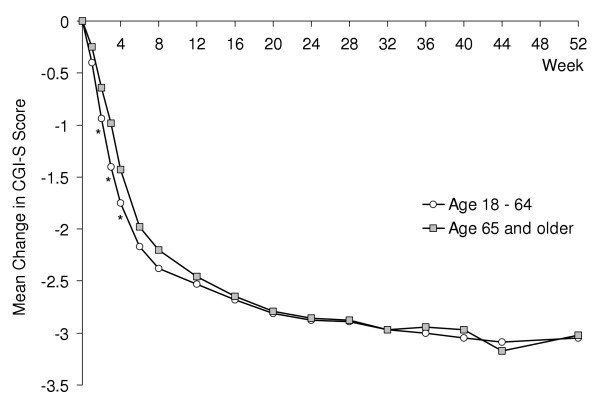
Comparison of mean change in CGI-Severity score for duloxetine-treated patients aged ≥ 65 years (n = 98) and age 18–64 years (n = 1121). * p ≤ .05 for between-group comparison.

A comparison of visitwise mean changes in CGI-S score between elderly patients (age ≥ 65, n = 101) and those patients in the study aged <65 years (n = 1178; Figure 1) revealed a somewhat more rapid onset of efficacy in younger patients, with differences between age groups being statistically significant at Weeks 2, 3 and 4. At subsequent visits the differences between age groups became progressively smaller, and mean changes were essentially equal at the study endpoint.

### Treatment discontinuation

The most common reasons for study discontinuation were adverse event (26.7%), personal conflict/other reasons (9.9%), and noncompliance (5.0%). The adverse events leading to discontinuation in >1.0% of enrolled patients at a duloxetine dose of 80–120 mg/d were somnolence (4.0%), dizziness (3.0%), diarrhea (2.0%), hypertension (2.0%), and vomiting (2.0%). Two-thirds of the discontinuations due to adverse events (18/27) occurred within 2 weeks of initiation of therapy.

### Serious adverse events

A total of 9 enrolled patients reported serious adverse events during the study. Most of these events were considered by the investigator to be unrelated to duloxetine exposure. The serious adverse events reported by more than 1 patient were hip fracture (2), and confusion (2), while there were single reports of agitation, angina pectoris, cerebrovascular disorder, coronary artery atherosclerosis, dementia, dizziness, hypomania, and myocardial ischemia. Individual occurrences were few, thus no clear temporal pattern of incidence of each event could be determined.

### Treatment-emergent adverse events

Treatment-emergent adverse events occurring in >5% of patients during the open-label therapy phase (Weeks 1 through 52) are summarized in Table 3. The incidence for these events during Weeks 1 to 8 and Weeks 9 to 52 are also listed in Table 3. During Weeks 1 through 52, adverse events reported by more than 10% of patients were dizziness, nausea, constipation, somnolence, insomnia, dry mouth, diarrhea, headache, and increased sweating. Over 75% of occurrences of these events were rated as mild or moderate in severity. The incidence of treatment-emergent adverse events was lower during the latter 44 weeks of the study (Weeks 9 to 52) than during the first 8 weeks. Each event with an incidence of at least 5% during Weeks 9 to 52 was also present at the same or higher rate during the first 8 weeks.

**Table 3:** Treatment-emergent adverse events^†^

**Event**	**Weeks 1–8, n (%)**	**Weeks 9–52, n (%)**	**Weeks 1–52, n (%)**
Nausea	29 (28.7)	0 (0.0)	29 (28.7)
Dizziness	27 (26.7)	5 (5.0)	31 (30.7)
Somnolence	22 (21.8)	1 (1.0)	23 (22.8)
Constipation	20 (19.8)	5 (5.0)	23 (22.8)
Dry mouth	16 (15.8)	4 (4.0)	18 (17.8)
Insomnia	15 (14.9)	8 (7.9)	22 (21.8)
Headache	11 (10.9)	6 (5.9)	16 (15.8)
Increased sweating	11 (10.9)	4 (4.0)	15 (14.9)
Diarrhea	11 (10.9)	6 (5.9)	17 (16.8)
Tremor	7 (6.9)	2 (2.0)	9 (8.9)
Anxiety NEC	7 (6.9)	3 (3.0)	10 (9.9)
Fatigue	7 (6.9)	4 (4.0)	9 (8.9)
Decreased appetite	7 (6.9)	1 (1.0)	7 (6.9)
Vomiting	7 (6.9)	3 (3.0)	10 (9.9)
Anorexia	6 (5.9)	3 (3.0)	8 (7.9)
Back pain	5 (5.0)	2 (2.0)	6 (5.9)
Abdominal pain upper	4 (4.0)	2 (2.0)	6 (5.9)

† Events with an occurrence > 5% in Weeks 1–52.

Rates of occurrence of other adverse events of importance in an elderly population were low: 2 patients experienced a fall, while there were single reports of syncope and postural hypotension. When analyzed by age group, patients aged 65 and older were found to report a significantly lower incidence of insomnia and headache than those patients aged <65 (Table 4). No other significant differences were observed between age groups.

**Table 4:** Treatment-emergent adverse events by age group^†^

	**N (%)**		
		
**Event**	**Age 18 – 64 (n = 1178)**	**Age ≥ 65 (n = 101)**	**p-Value**
Nausea	406 (34.5)	29 (28.7)	.274
Insomnia	378 (32.1)	22 (21.8)	.034
Headache	373 (31.7)	16 (15.8)	<.001
Somnolence	358 (30.4)	23 (22.8)	.114
Dry mouth	282 (23.9)	18 (17.8)	.180
Dizziness	267 (22. 7)	31 (30.7)	.085
Constipation	250 (21.2)	23 (22.8)	.705
Increased sweating	177 (15.0)	15 (14.9)	1.00
Anxiety	176 (14.9)	10 (9.9)	.188
Diarrhea	157 (13.3)	17 (16.8)	.363
Fatigue	125 (10.6)	9 (8.9)	.735

† Events with an occurrence > 10% in Weeks 1–52.

### Cardiovascular profile

Mean changes from baseline to last observation for standing and supine systolic and diastolic blood pressures were less than 2 mm Hg and not significantly different from zero: supine systolic BP -1.5 mm Hg (p = .364), supine diastolic BP -1.8 mm Hg (p = .141), standing systolic BP -1.9 mm Hg (p = .269), standing diastolic BP -0.1 mm Hg (p = .907). Using repeated measures analysis, mean changes in blood pressure were <4 mm Hg at every visit from baseline to endpoint.

**Figure 2:**
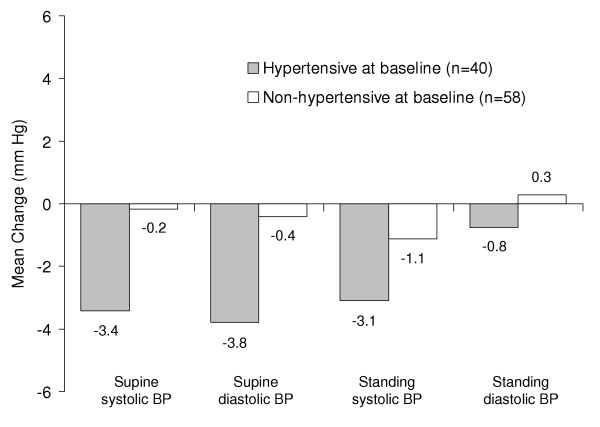
Mean change from baseline to endpoint in blood pressure (mm Hg) for baseline hypertensive and non-hypertensive patients aged ≥ 65 years receiving duloxetine (80–120 mg/d). p > .10 for all between-group comparisons.

A mean change analysis was utilized to compare blood pressure in patients who were hypertensive (n = 40) versus non-hypertensive (n = 58) at baseline. Baseline hypertensive patients exhibited small mean decreases (<4 mm Hg) in both standing and supine systolic and diastolic blood pressures from baseline to endpoint, while patients who were not hypertensive at baseline demonstrated mean changes in these same measures of 0.3 to -1.1 mm Hg (Figure 2).

Mean baseline-to-endpoint increases were observed for supine pulse (mean change = 1.6 bpm, p = .105) and standing pulse (mean change = 1.1 bpm, p = .338) but these values did not differ significantly from zero.

Rates of occurrence of potentially clinically significant (PCS) values for systolic and diastolic blood pressures were generally low. The incidence of PCS low standing systolic blood pressure was 5/96 (5.2%), while all other assessed blood pressure and pulse readings had incidences of PCS values <2.5%.

There were no significant changes in cardiac intervals detected by ECG. Mean changes from baseline to last observation were: PR -3.3 msec (p = .363), QRS -2.5 msec (p = .420), QT 5.0 msec (p = .730), and QTcF 6.2 msec (p = .553). No patient experienced a PCS QTcF value during the course of the study.

### Body weight

**Figure 3:**
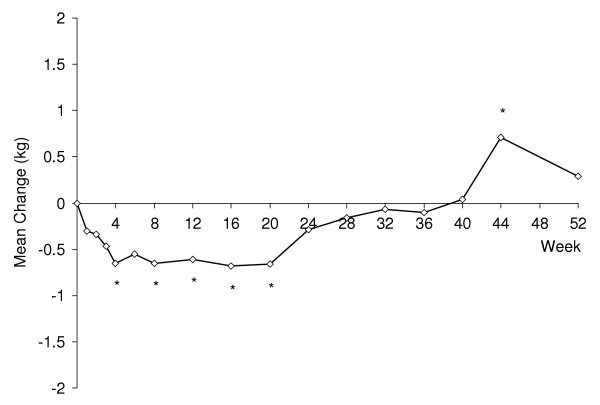
Mean change in weight (kg) for duloxetine-treated patients aged ≥ 65 years (dose 80–120 mg/d, n = 98). *p ≤ .05 from t-test for mean change.

After 52 weeks of treatment, the mean change in weight from baseline to last observation was -0.1 kg (p = .741), while a mean weight change of +0.3 kg was determined using MMRM analysis (p = .386 for t-test for mean change at endpoint; Figure 3). Mean changes in weight at early visits were negative (weight loss), mean changes at intermediate visits were near zero, while mean changes at later visits were positive (weight gain). A total of 3/98 patients (3.1%) experienced PCS weight loss while 6/98 (6.1%) reported a PCS weight gain (PCS weight change is defined as a change of ≥ 10% of baseline body weight). The 3 patients displaying PCS weight loss had baseline body mass indices (BMI) of 24.9, 28.5 and 32.1, while those experiencing weight gain had BMIs at baseline ranging from 19.9 to 26.7.

### Laboratory analytes

Statistically significant mean changes were observed in some laboratory analytes. Despite the statistical significance, the magnitudes of the mean changes were generally small and not considered clinically relevant in light of the low incidence of potentially clinically significant (PCS) values.

### Discontinuation-emergent adverse events

All patients who proceeded past Week 52 received no study drug for 2 weeks until Week 54 via abrupt discontinuation (no taper). Discontinuation-emergent adverse events occurring in ≥ 5% of patients were dizziness (8.9%), anxiety (7.9%), headache (5.0%), and insomnia (5.0%).

## Discussion

The current analysis focused upon 101 depressed patients aged 65 years and older who received long-term, open-label treatment with duloxetine (80 mg/d or 120 mg/d). Efficacy was demonstrated on all assessed outcome measures, both clinician- and patient-rated. Highly significant improvements were seen in both patient- and clinician-rated depression and health outcome scales (CGI-S, HAMD_17_, BDI-II, PGI-I, SDS) at all visits. By way of comparison, significantly greater improvements for duloxetine compared with placebo were observed in HAMD_17 _total score, HAMD_17 _subscales and CGI-S score in two 9-week, placebo-controlled studies of duloxetine (60 mg once daily (QD)) in patients aged 55 years and older [12].

Onset of efficacy is an important consideration in antidepressant trials, but in the absence of a placebo arm it is especially difficult to define and assess [23]. However, the significant improvements from baseline in CGI-S and PGI-I scales at Weeks 1 and 2 are consistent with results from double-blind, placebo-controlled trials in which duloxetine demonstrated significant superiority over placebo as early as Week 1 on core emotional symptoms of depression (HAMD_17 _Maier subscale), and global improvement (CGI-S scale) [8]. It has also been suggested that treatment response may be slower and/or less robust in an elderly population compared with a younger cohort [24]. Indeed, in the present study a more rapid onset of efficacy was observed in duloxetine-treated patients aged 18–64 when compared with those patients aged ≥ 65. However, the magnitude of treatment differences between age groups progressively diminished and was not significant at any visit after Week 4. This result may have substantial clinical relevance for long-term treatment. It suggests that, although those patients aged 65 and older may exhibit a somewhat less rapid onset of antidepressant action than a younger cohort, elderly patients are able to reach and sustain a level of depressive symptom improvement equal to that observed in younger patients.

In this study, observed case response and remission rates following 6 weeks of open-label duloxetine therapy (62.9% and 41.4%, respectively) were comparable to the response and remission rates (52.8% and 44.1%, respectively) observed in older patients in two 9-week double-blind, placebo controlled trials of duloxetine (60 mg QD) [12]. Furthermore, remission rates at 52 weeks in the present study were only slightly less than response rates (72.3% and 89.4%, respectively), implying that those patients who responded had a high probability of achieving complete symptom resolution. A growing body of evidence suggests that remission, rather than response, should be the goal of antidepressant treatment [25]. Responders who do not remit may have appreciable residual symptomatology, and patients with residual symptoms have been found to be at higher risk for relapse or recurrence [26]. Given the high rates of relapse and recurrence observed among elderly patients, achievement of remission assumes an added degree of importance.

In light of the recommendation that elderly patients receive at least 12–18 months of antidepressant therapy [27], the long-term safety and tolerability of these medications are of considerable importance. Duloxetine was safely administered and well-tolerated in this long-term study. While the discontinuation rate due to adverse events (26.7%) was somewhat higher than that observed in older patients (aged ≥ 55) in two 9-week, placebo-controlled trials of duloxetine 60 mg QD (21.0%), the difference in these rates suggests that few patients stopped taking medication during the periods associated with continuation and maintenance treatment. The discontinuation rate is also comparable to that observed in a 54-week study of fluoxetine in elderly patients [28], and is only slightly higher than that obtained from a meta-analysis of acute-phase (≤ 8 week) trials of SSRIs in elderly patients (14.3%–22.8%) [29]. Given the one-year duration of this study, and the administration of duloxetine at the upper end of its studied dose range (80–120 mg/d) throughout the trial, the long-term tolerability of duloxetine in elderly patients appears to be comparable to that of SSRIs.

The incidence and pattern of treatment-emergent adverse events during Weeks 1 to 8 of this study were generally similar to those observed in acute-phase, placebo-controlled trials in older patients [12]. The most frequently reported adverse events were nausea, dizziness, somnolence, constipation, and dry mouth. Most of the events were either mild or moderate in severity and transient in nature. During the last 44 weeks of the study, no adverse event occurred in more than 8% of the patient population and the incidence of each specific event was generally lower in the entire period from Weeks 9–52 than in the initial 8 weeks of the study. Thus, patients who tolerated duloxetine during the early period of the trial were likely to tolerate long-term dosing.

Administration of medication to elderly patients necessitates consideration of the physiological changes which accompany aging. Such changes can result in substantial differences in adverse event profiles between older and younger patient populations [30]. In this study, comparisons between age groups (18–64 years vs. ≥ 65 years) of the most commonly reported treatment emergent adverse events revealed significant differences only in the rates of insomnia and headache. Furthermore, in each of these cases the higher rates were observed in the younger age group. In the absence of a placebo control arm these results must be viewed with an appropriate degree of caution, but they provide an indication that the adverse event profile for duloxetine in the elderly may be similar to that observed in younger patients.

Antidepressants with benign cardiovascular profiles may be particularly suitable for the treatment of an elderly population, in which heart disease is more prevalent than in younger patients [31]. In this study, duloxetine-treated patients exhibited small (less than 2 mm Hg) mean changes in blood pressure from baseline to endpoint and low rates of PCS blood pressure values. Furthermore, those patients with baseline hypertension demonstrated a mean decrease in blood pressure compared with normotensive patients. Consistent with the profile of duloxetine as a NE reuptake inhibitor, small mean increases (less than 2 bpm) were observed in heart rate. Mean changes in corrected QT interval were small and not significantly different from zero, suggesting duloxetine did not prolong QT intervals. Collectively, these data indicate that duloxetine exhibits a favorable cardiovascular profile in elderly patients.

Weight change is an important consideration in older patients being treated with antidepressants [32], especially during long-term treatment. Following 52 weeks of open-label duloxetine treatment, mean change in weight from baseline to last observation was -0.1 kg. Repeated measures analysis was used to derive a longitudinal profile of weight change. This revealed a small (<1 kg) decrease in weight at early visits, consistent with the weight change of -0.2 kg observed in older patients in two 9-week acute trials of duloxetine [12]. However, mean changes at intermediate visits approached zero, while mean changes at the last 2 visits were positive (weight gain). A total of 3/98 patients (3.1%) reported a PCS weight loss while 6/98 (6.1%) reported a PCS weight gain. By way of comparison, a recent study of weight change among depressed nursing facility residents aged >65 who received ≥ 6 months of antidepressant treatment found rates of clinically important weight loss and weight gain (defined as ≥ 10% change in body weight or Minimum Data Set-Plus weight loss or weight gain marker) of 14.7% and 14.4%, respectively [33].

It is important to consider all of the safety findings described here in light of the dosing and design requirements of the study. The doses used in this open-label study were up to 2-fold greater than the once-daily 60 mg duloxetine dose which has been shown to provide robust efficacy in older patients in placebo-controlled trials [12]. The dosing and other design features of the study (e.g. the intensive visit schedule) were specifically included to maximize the probability of uncovering adverse reactions to duloxetine. Furthermore, no special dosing guidelines were implemented for these elderly patients. While lower doses of many antidepressants are recommended in the elderly [34], especially due to concerns of adverse events among the TCAs, this can lead to the use of subtherapeutic doses and corresponding reductions in efficacy [35]. In this study, however, the comparable adverse event profiles observed for elderly and younger age groups suggest that a duloxetine dose which has been shown to provide robust efficacy may be safely administered in depressed patients regardless of age. Only in particularly sensitive elderly patients may dosing adjustments be required.

## Conclusions

Results from this open-label study of depressed patients aged 65 and older suggest that duloxetine is safe and well tolerated in long-term use. Statistically significant and clinically relevant improvements in all assessed efficacy measures were observed at each patient visit. Furthermore, the efficacy and adverse event profile of duloxetine appears to be comparable in older (age ≥ 65) and younger patients (age 18–64). These results, together with those obtained from acute phase, double-blind, placebo-controlled trials, support the efficacy of duloxetine in the treatment of major depression in older patients.

## Competing interests

Drs. Wohlreich, Mallinckrodt, and Watkin are employees of Eli Lilly and Company. Dr. Hay was employed by Eli Lilly and Company at the time of the study.

## Authors' contributions

MMW, CHM, JGW, and DPH participated in interpretation of data and drafting of the manuscript. CHM carried out the statistical analyses. All authors read and approved the final manuscript.

## Pre-publication history

The pre-publication history for this paper can be accessed here:


